# Has it become increasingly expensive to follow a nutritious diet? Insights from a new price index for nutritious diets in Sweden 1980–2012

**DOI:** 10.3402/fnr.v59.26932

**Published:** 2015-04-08

**Authors:** Andreas Håkansson

**Affiliations:** Food and Meal Science, School of Education and Environment, Kristianstad University, Kristianstad, Sweden

**Keywords:** diet cost, nutritional recommendations, nutritional intake, linear programming, goal programming, socioeconomic status, vitamin D, fruit and vegetables

## Abstract

**Background:**

Health-related illnesses such as obesity and diabetes continue to increase, particularly in groups of low socioeconomic status. The increasing cost of nutritious food has been suggested as an explanation.

**Objective:**

To construct a price index describing the cost of a diet adhering to nutritional recommendations for a rational and knowledgeable consumer and, furthermore, to investigate which nutrients have become more expensive to obtain over time.

**Methods:**

Linear programming and goal programming were used to calculate two optimal and nutritious diets for each year in the interval under different assumptions. The first model describes the rational choice of a cost-minimizing consumer; the second, the choice of a consumer trying to deviate as little as possible from average consumption. Shadow price analysis was used to investigate how nutrients contribute to the diet cost.

**Results:**

The cost of a diet adhering to nutritional recommendations has not increased more than general food prices in Sweden between 1980 and 2012. However, following nutrient recommendations increases the diet cost even for a rational consumer, particularly for vitamin D, iron, and selenium. The cost of adhering to the vitamin D recommendation has increased faster than the general food prices.

**Conclusions:**

Not adhering to recommendations (especially those for vitamin D) offers an opportunity for consumers to lower the diet cost. However, the cost of nutritious diets has not increased more than the cost of food in general between 1980 and 2012 in Sweden.

Overweight, obesity, cardiovascular disease, and diabetes – indicators of unhealthy dietary habits in a population – continue to increase in many parts of the world (1). In Sweden, the prevalence of obesity more than doubled between 1980 and 2012 (2). There are also large inequalities in these diet-related illnesses linked to socioeconomic status (SES); low SES increases the risk for overweight and obesity (3–7), and similar trends can be seen for blood pressure (8) and cardiovascular disease (9).

Diet recommendations are regularly published by national and international health authorities with the intention of decreasing diet-related morbidity and loss of quality of life. Despite these good intentions, recommendations are not always followed, and unhealthy eating prevails (10, 11). Failure to comply with diet recommendations is not evenly spread throughout society. Higher-income families consume more fruits and vegetables and whole grains (9, 12–15). Consumers with higher SES have also been associated with higher diet quality and variability, especially in relation to micronutrients, such as minerals and vitamins (16, 17). The well-established difference in dietary patterns, as well as the degree to which dietary recommendations are met, can be an important factor explaining socioeconomic inequality in health (16, 18). Several studies have suggested that more nutrient-dense foods, less energy-dense foods, and foods adhering to nutritional recommendations are more expensive per unit of energy (19–22). Furthermore, some researchers have argued that the price of nutritious foods and diets is increasing at a faster rate than less nutritious alternatives (21, 23). However, other studies have shown that healthy foods, such as fruits and vegetables, did not increase more in price than unhealthy alternatives, such as snacks, over a period of 25 years (24) and that there is no significant difference in the price increases of what are defined as ‘core’ (necessary, healthy foods) versus ‘noncore’ foods (25). Because of these opposing results, a consensus on the relative increase in the price of healthier foods or diets has not yet been reached (26). All of these previous studies focused either on food in general or on a predefined food bundle. At least for longer periods of time, consumers could adjust consumption to price changes. Furthermore, for nutritionists and policy makers interested in influencing consumption, it is interesting to understand to what extent a rational and knowledgeable consumer could rearrange his or her consumption to avoid price increases and still follow a nutritional diet, because this would set the baseline for how much of the current problem could be solved through information or education.

Linear programming (27, 28) and nonlinear optimization (29–32) (often referred to as ‘goal programming’ in the nutrition literature) are promising tools for systematic investigations of the relation between food prices and diet nutritional quality (29, 30, 33, 34). They provide an approach to investigating the diet cost of a rational consumer with full knowledge of alternatives and consequences under explicitly stated assumptions. Furthermore, they provide – through shadow price analysis (35) – a tool for assessing how much each nutrient contributes to the evolution of a nutritious diet, a factor that has not been investigated in prior studies.

In this study, we set out to develop a set of price indexes, using linear and goal programming, to investigate how the cost of nutritious diets has developed over time. We undertook this project in order to answer the following research questions: (1) Did the cost of a diet fulfilling the current nutrition recommendations increase more or less than the consumer price index (CPI) for food in Sweden between 1980 and 2012? (2) How did the implicit cost of individual nutrient recommendations contribute to these changes?

## Materials and methods

### Linear programming and goal programming

Linear programming and goal programming for nutritional analysis have been comprehensively described elsewhere (29, 31, 32). In short, they are techniques for selecting an optimal combination of foods under a set of nutritional constraints. Each optimization consists of a set of decision variables: the amount of each included food; a set of constraints, typically on the absolute total daily intake of a set of nutrients; and an objective function to minimize. The solution given by the optimization is the set of decision variable values that minimizes the objective function under the given constraints. In linear programming, the objective function is a linear combination of the decision variables, typically the total diet cost (27, 28, 33, 34). In goal programming, the objective function can be a nonlinear function of the decision variables, typically the relative deviation from an observed average consumption pattern (29, 30, 32).

Two diet optimization models were used in this study to model different consumer behavior. A linear programming minimum price (MP) model was used to calculate the minimum cost of a food meeting a set of nutritional and cultural constraints, and a minimum deviation goal programming (MD) model was used to calculate the diet with minimum relative deviation from the current consumption (disregarding price). The MP model (27, 33, 34) represents a highly price-sensitive consumer who is willing to make substantial changes to the diet in order to adhere to recommendations at the lowest cost, whereas the MD model (29, 30, 32) represents a price-insensitive consumer who adheres to nutritional recommendations by choosing a diet that differs as little as possible from the average consumption of the population.

In both models, the decision variables consisted of 101 predefined food items, identified from the detailed categories of a large national dietary survey (11).

A linear programming algorithm was used for the MP model. Three nonlinear optimization solvers were used and compared (interior point, active set, and sequential quadratic programming) for the MD model. There was a strong correlation between the prices obtained by the different solvers (*r*>0.996). The solver arriving at the lowest cost while fulfilling all constraints (interior point in all cases) was used throughout the analysis. All algorithms were used as implemented in MATLAB 2013b (MathWorks, Natick, MA). The MP and MD methods are compared and summarized in Table 1.

**Table 1:** Summary of the two optimization methods

	Minimum price (MP) model	Minimum difference (MD) model
Objective function	Minimize total price $x_{MP}^{*}=\begin{array}{c} \min\\ x \end{array}\sum_{i=1}^{I}p_{i}\cdot x_{i}$ *p*_*i*_ is the price of food *i=*1…*I x*_*i*_ is the mass per day consumed of food *i*.	Minimize relative deviation from average intake: $x_{MD}^{*}=\begin{array}{c} \min\\ x \end{array}\sum_{i=1}^{I}\frac{|x_{i}-X_{i}|}{X_{i}}$ *X*_*i*_ is the observed average consumption of food *i* according to dietary surveys.
Constraints	1. Nutritional constraint: nutrition recommendations from Table 2 2. Cultural constraint: upper limitations corresponding to the 95th percentile of current consumption of the food groups in Table 3 3. Cultural constraint: upper limitation on each individual food component: mean plus three standard deviations of actual intake	
Decision variables	Consumed amounts of (*I* =) 101 different foods	
Algorithm	Linear programming	Constrained nonlinear optimization (interior-point algorithm)

### Nutritional constraints

Both models (MP and MD) were subjected to nutrition constraints based on the recommended daily intake as stated by the present Nordic Nutrition Recommendations (NNR) (36) for individuals aged 31–60. The full set of constraints expressed as daily intake can be seen in Table 2 and includes constraints on energy, minimum intake of dietary fiber, 18 nutrients, and six intervals of energy percentages. Nutrients with tolerable upper intake levels according to the NNR (36) were constrained to maximal daily intake in order to further ensure realistic levels in the optimum diet. In addition, the total intake of fruits and vegetables [defined according to the recommendation of the Swedish National Food Agency to include vegetables, root vegetables (excluding potatoes), fruits, berries, and juices] was required to be above the recommended 500 g per day (37) for both models. The selection of included nutrients was based on the NNR fact sheet published by the Swedish National Food Agency (37). The lower bound for energy intake corresponds to low activity (sedentary lifestyle, PAL 1.6) and the upper corresponds to high activity (active lifestyle, PAL 1.8) (36).

**Table 2:** Nutritional constraints on daily intake used in the MD and MP models

	Women 31–60 years		Men 31–60 years	
	
Nutrient	Min	Max	Min	Max
Energy (kcal)	2,100^a^	2,630^b^	2,630^a^	2,960^b^
Fat in% of TE	25%^c^	40%^c^	25%^c^	40%^c^
Carbohydrates in% of TE	45%^c^	60%^c^	45%^c^	60%^c^
Protein in% of TE	10%^c^	20%^c^	10%^c^	20%^c^
SFA in% of TE	–	10%^c^	–	10%^c^
PUFA in% of TE	5.0%^c^	10%^c^	5.0%^c^	10%^c^
MUFA in% of TE	10%^c^	20%^c^	10%^c^	20%^c^
Fiber (g)	30^d^	–	30^d^	–
Calcium (mg)	800^d^	2,500^e^	800^d^	2,500^e^
Folate (mg)	300^d^	1,000^e^	300^d^	1,000^e^
Iodide (µg)	150^d^	600^e^	150^d^	600^e^
Iron (mg)	15^d^	25^e^	9^d^	25^e^
Magnesium (mg)	280^d^	–	350^d^	–
Niacin equivalents (NE^f^)	14^d^	–	18^d^	3,000^e^
Phosphorus (mg)	600^d^	3,000^e^	600^d^	3,000^e^
Potassium (mg)	3,100^d^	–	3,500^d^	–
Riboflavin (mg)	1.2^d^	–	1.5^d^	–
Selenium (µg)	50^d^	300^e^	60^d^	300^e^
Sodium (mg)	–	2,400^e^	–	2,400^e^
Thiamine (mg)	1.1^d^	–	1.3^d^	–
Vitamin A (RE^g^)	700^d^	3,000^e^	900^d^	3,000^e^
Vitamin B12 (µg)	2.0^d^	–	2.0^d^	–
Vitamin B6 (mg)	1.2^d^	25^e^	1.5^d^	25^e^
Vitamin C (mg)	75^d^	1,000^e^	75^d^	1,000^e^
Vitamin D (α-TE^h^)	10^d^	100^e^	10^d^	100^e^
Vitamin E (µg)	8.0^d^	300^e^	10^d^	300^e^
Zink (mg)	7.0^d^	25^e^	9.0^d^	25^e^

PUFA: polyunsaturated fatty acids; MUFA: monounsaturated fatty acids; SFA: saturated fatty acids; TE: total energy. Reference data from Nordic Nutrition Recommendations (NNR) (36).

a PAL 1.6

b PAL 1.8

c NNR recommendations

d recommended daily intake

e tolerable upper intake

f niacin equivalents: 1 niacin equivalent=1 mg niacin=60 mg tryptophan

g retinol equivalents: 1 retinol equivalent=1 µg retinol=12 µg β-carotene

h α-tocopherol equivalents: 1 α-tocopherol equivalent=1 mg RRR-α-tocopherol.

### Dietary patterns for cultural constraints

Dietary patterns for Sweden were obtained from national self-reporting surveys conducted in 2010–2011 (11), 1997–1998 (38), and 1989 (39). The surveys differ somewhat in how food intake is categorized and reported. The food items included were divided into 25 categories, as shown in Table 3, for standardization between years. These categories correspond closely to the ones used in the 2011 survey. The average daily intake for each food item for years other than the ones surveyed was estimated from linear interpolation between surveyed years. Intake before 1989 and after 2011 was assumed to be equal to the closest available survey. This procedure resulted in an average intake of each of the 101 foods for each year from 1980 to 2012.

**Table 3:** Food groups, corresponding consumer price index (CPI) (41), and the obtained optimal 2011 intake from MP and MD diets for a woman (aged 31–60 years) as compared to actual intake (11)

Food group	CPI food price index	MP diet 2011 (% of actual intake)	MD diet 2011 (% of actual intake)
Spreads	Dietary fats and oils	291	100
Cheese	Milk, cheese, and eggs	0	100
Milk, fermented milk, and yogurt	Milk, cheese, and eggs	229	101
Bread	Bread and cereal	34	147
Potato	Vegetables	85	104
Root vegetables	Vegetables	373	143
Vegetables	Vegetables	94	151
Fruit and berries	Fruits	213	129
Juices	Soft drinks and juices	0	135
Pasta	Bread and cereals	385	126
Meat	Meat	28	124
Egg	Milk, cheese, and eggs	364	294
Fish and shellfish	Fish	56	235
Sausages	Meat	0	107
Buns, cookies, and cakes	Sweets and ice cream	0	115
Ice cream	Sweets and ice cream	475	100
Sweet soups and desserts	Sweets and ice cream	0	100
Preserves	Food	0	100
Soft drinks	Soft drinks and juices	0	100
Candy	Sweets and ice cream	0	102
Sugar and sweeteners	Food	700	101
Coffee and tea	Coffee, tea, and cocoa	0	100
Alcoholic beverages	Alcoholic beverages	0	107
Nuts and savory snacks	Sweets and ice cream	85	441
Cereals, rice, and porridges	Food	160	149

Both models (MP and MD) were constrained by the requirement that no food group be consumed at levels above the 95th percentile of the actual intake of the respective food group according to the surveys. Furthermore, no single food item was allowed to be consumed above the mean value plus three standard deviations of the actual consumption. (For surveyed foods where the standard deviation was not given, this was estimated by assuming the food to have the same coefficient of variation as the average of all foods.) These constraints are relatively mild and were imposed to exclude the most culturally unfeasible diets.

### Selection of food items, nutritional composition, and price data

Nutritional composition for each of the 101 food items was obtained from the Swedish Food Agency's database (40).

Current food prices for all the food items were obtained as consumer prices from a survey of Swedish online grocery stores (Cooponline and Mathem, prices obtained May 2014 for Stockholm). Most food items are available from different brands and have different packaging, origin, and quality. The choice of specific products was based on the popularity of the items as ranked by the online food grocer.

Representative historical prices for each of the 101 foods were not available and were estimated by deflating the present prices with the CPI data for the corresponding food group (41). Statistics Sweden carries CPI data on 16 groups of foods. The linking of these 16 food CPI groups to the 25 food groups of the nutritional survey can be seen in Table 3.

### Methodology to validate deflated prices and average intake

The deflation-based price estimations per food group needed to be validated to ensure that the optimizations described reliable price levels. For this, the national household food expenditure was compared to the estimated actual expenditures based on the suggested deflation methodology. The estimated nominal price of the 101 food items together with the estimated average intake of these for each year was used to estimate the actual daily food expenditure per capita during the period. These estimations were compared to the per capita national Swedish household food expenditure from Statistics Sweden (42) in order to validate the estimated data. However, an unadjusted comparison is not a meaningful comparison, because the national dietary surveys do not show the same general volumetric per capita increase in food consumption as the food expenditure data (11, 42). This indicates that the purchased per capita food volume has increased faster than the per capita consumption. In order to obtain suitable validation data, the total per capita food expenditure was normalized to this volumetric increase [using data from Statistics Sweden (42)] before comparing it to the estimated food expenditures. The obtained validation data is referred to as the ‘volume-adjusted per capita food expenditure’ in the study.

### Methodology to calculate the price index for nutritious foods

For each year in the interval 1980–2012, two MP (nutritional recommendations for men and women respectively) and two MD optimizations were run according to Table 1. Each optimization was based on the estimated average intake and deflated prices for that year. A minimal price nutritious food index (MPNFI) was constructed from the average of the price of the MP optimal bundle for men and women normalized to the average value of the first year (1980). The minimal deviation nutritious food index (MDNFI) was formed analogously based on MD model optimizations.

### Shadow price analysis

Although underutilized in nutritional optimization, shadow prices are often used in economics and operation analysis to estimate how costly each of the constraints in a linear programming optimization are. Formally, the shadow price equals the Lagrangian multiplier in the corresponding optimization problem and describes the incremental increase in the optimal cost when each constraint is increased by an infinitesimal amount (43). For the MP model, the shadow price describes how much each nutritional recommendation contributes to the minimum diet cost for a highly price-sensitive rational consumer, and thus it offers a method of studying which nutrients are the most expensive to obtain (35).

## Results

### Validity of price estimation procedure

**Fig. 1:**
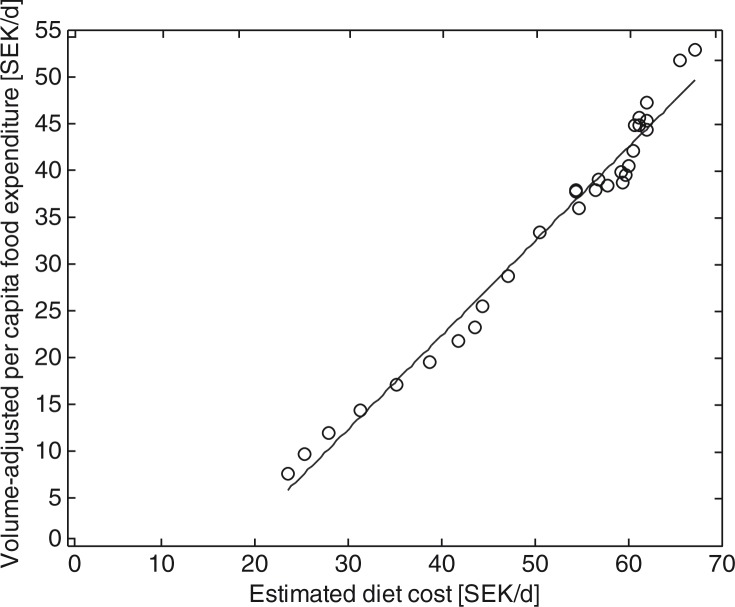
Comparison of estimated diet cost for each year 1980–2009 compared to volume-adjusted per capita food expenditures for validation of the price estimation method. Dashed line is the linear least-square fit to the data.

Accurate calculation of price indexes requires that estimations of prices and consumed quantities are sufficiently reliable. The estimated total food expenditure for an individual using the proposed method is highly correlated with the volume-adjusted per capita food expenditure (*r*=0.99; see Fig. 1) and has a near unity proportionality constant (the slope of the least square linear regression model in Fig. 1 is 0.97). However, it systematically overestimates the absolute cost by approximately 18 SEK/day (intercept in the regression model in Fig. 1). Thus, neither the MP model nor the MD model should be used to estimate absolute price levels; however, they are expected to reliably describe price variations over time and are thus suitable for forming a price index.

### Diet cost and composition

MD diets are generally more expensive than MP diets as a result of the higher emphasis on cultural adherence. For 2011, for men (women) the MP diet cost was 41 SEK/day (33 SEK/day) and the MD diet cost was 96 SEK/day (95 SEK/day).

Diet composition for MP and MD diets as of 2011 can be seen in Table 3. The MP diet differs significantly from the average intake. It contains more of some food groups that are generally considered healthy, such as root vegetables, fruits, and berries, but also a much higher intake of ice cream and sugar (although from an initial low level). The increase in energy- and nutrient-dense foods is expected because the national intake shows energy and nutrient intake per day below the constrained level (11). The MD diet, with more emphasis on cultural constraints, is closer to the average intake but promotes a higher intake of nuts, fish, and vegetables – food groups that are generally seen as parts of a healthy diet.

### Price indexes

**Fig. 2:**
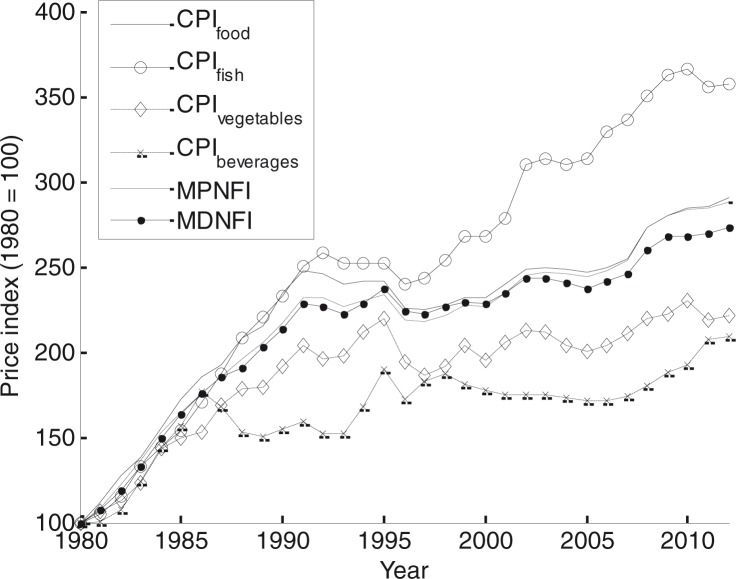
Minimum price nutritional food index (MPNFI) and minimum deviation nutritional food index (MDNFI) compared to national food CPI of foods in general and three food groups.

Both the MPNFI and the MDNFI follow the food CPI closely between 1980 and 2012. Both indexes increased slightly slower than the food CPI (see Fig. 2). For the MDNFI the difference is 5%. The difference is less than 1% for the MPNFI. CPI data for the food group with the highest (fish and shellfish), lowest (nonalcoholic beverages), and second-lowest (vegetables) price increase between 1980 and 2012 have been included to show the variability in the underlying CPI data.

### Shadow price analysis

The shadow prices show which constraints are actively limiting the solution. For the MP model, the shadow prices could be directly translated to the cost of each constraint. Using the nutrient recommendations for women, the highest shadow prices for the 2011 diet are obtained for the minimum recommended intake of vitamin D (0.56 SEK/µg/day), iron (0.40 SEK/mg/day), fruits and vegetables (0.022 SEK/g/day), and energy (0.010 SEK/kcal/day). The limiting nutrients for men are similar, with the exception that iron is replaced by selenium and the order altered: selenium (0.27 SEK/µg/day), vitamin D (0.031 SEK/µg/day), fruits and vegetables (0.022 SEK/g/day), and energy (0.013 SEK/kcal/day). This list should be interpreted as the order of what nutritional requirements are the most costly to comply with under the prevailing prices and nutritional recommendations for a highly price-sensitive rational consumer.

**Fig. 3:**
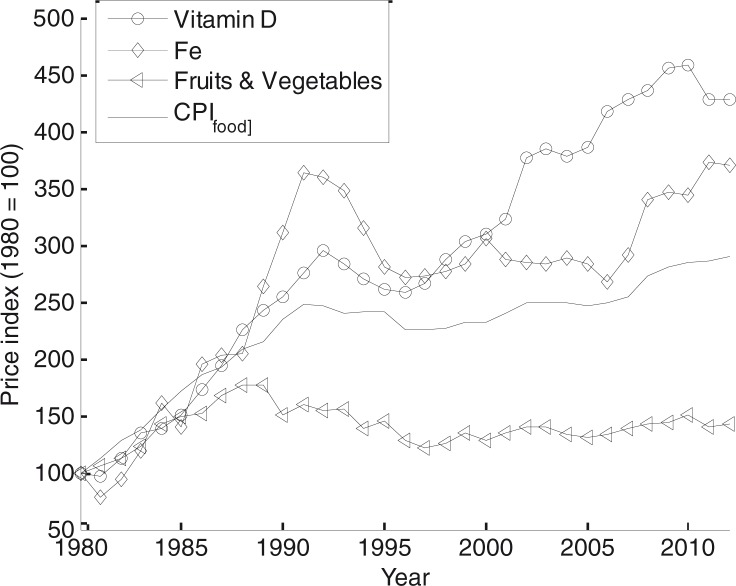
Shadow price indexes for the limiting nutrients for women.

The cost of meeting the most costly recommendations change over time. It is measured by MP model shadow price index in 1980–2012 (using 1980 as the base year). Significant variations can be seen between the different nutrients. The shadow prices for vitamin D and iron increase at a rate significantly higher than overall indexes, whereas those for fruits/vegetables increase at a lower rate (see Fig. 3). Shadow prices for men show similar results, with a large increase in the relative prices of the vitamin D recommendation and a modest increase in the price of the fruits/vegetables recommendation.

## Discussion

### The increasing price of nutritious food

In summary, the results show that the cost of a nutritious diet did not increase more than the food CPI in Sweden between 1980 and 2012. This result is far from obvious because there is a large variability between the CPI of the different food groups, as seen in Fig. 2. Figure 2 also shows that not all food groups that are considered nutritious (e.g. fish and vegetables) follow the same trend [cf. (24)]. The food CPI in Sweden has increased slower than the overall CPI during the last decades (41). Thus, compared to other goods, food has a relatively lower price today than 30 years ago. However, the food CPI bundle is chosen based on actual consumption and does not say anything about the nutritional value of the included foods. The MPNFI and MDNFI are attempts to form price indexes of nutritious foods; in particular, they are two indexes describing how costly it would have been for a rational consumer to fulfill the present nutritional recommendations based on prices and preferences from 1980 and onward.

The absolute diet cost for the MD and MP diets can be compared to the per capita food expenditure in Sweden, 58 SEK/day in 2011 (11, 42). Thus, the MP diet at 37 SEK/day (average over men and women) is lower than the actual expenditure, whereas the MD diet cost at 96 SEK/day is higher. The cost of the nutritious diets are thus comparable to actual expenditure; however, absolute costs calculated from the MD and MP models should not be over interpreted, because the validation procedure shown in Fig. 1 indicates an underestimation.

The results of this study can be compared to those of Monsivais et al. (21), which showed that prices of foods with a high nutrient density increased faster than those of foods with lower nutrient density between 2004 and 2008 in the United States. It can also be compared to the results of Harrison et al. (23), which showed that the price of a predefined basket of commonly consumed foods containing a high percentage of recommended nutrient intake increased faster than the CPI for food in Australia between 1998 and 2006. These results are not contradictory, because they investigate different aspects of the phenomenon owing to methodological differences. This difference is more clearly seen in terms of what assumptions they make on how consumers choose foods to attain a nutritious diet. Monsivais et al. (21) showed that a consumer choosing a representative sample – e.g. by choosing at random – of all available nutrient-dense foods would experience a higher price increase than consumers choosing a representative sample among nutrient-poor foods. This price increase could act as an incitement for such a consumer to switch from nutrient-dense to nutrient-poor foods. Assuming that the predefined bundle is representative of nutrient-rich and culturally acceptable diets, Harrison et al. (23) showed that a consumer keeping to the same consumption pattern would experience a higher cost increase if this bundle adhered to recommendations than if it simply followed average consumption. The present study shows that the price increase experienced by a rational consumer able to change consumption in order to keep to recommendations and minimize either food expenditures (MP model) or deviation from the average intake of the population (MD model) is not faster than that of food in general. The three methodologies are not expected to give similar results. For instance, the fact that higher nutrient density foods on average increase more in price than low nutrient density foods does not have to influence the cost and choice of the thrifty consumer if there are still enough culturally acceptable and nutritious foods that are exceptions to the general rule. This can be seen from the data in the present study. Despite the significant increase in the price of fish, the shadow price for obtaining the recommended energy percentage of polyunsaturated fatty acids remained low (<10^−6^ SEK/%/day) throughout the period, most likely as a result of the lower-than-average increase in the price of vegetable oils (41). Thus, rational consumers can obtain their polyunsaturated fatty acids by consuming less fish and more vegetable oil when prices change (assuming that the other necessary nutrients present in fish and shellfish can also be obtained from other sources).

The studies taken together indicate that, although some nutritional food groups do increase in price, it is, at least in the Swedish context, possible for consumers to avoid this increase in cost by making rational and knowledgeable choices.

### The cost of individual requirements

The shadow price analysis shows that adhering to the nutritional recommendations does increase the diet cost even for rational and knowledgeable consumers. The recommendations that are the most costly are the minimal intake of vitamin D, iron (for women only), selenium (for men only), and fruits and vegetables. Shadow prices differ between men and women because of differences in nutritional requirements; from the large differences in iron, selenium, and vitamin D, for example, it can be seen that different nutrients are costly to obtain for men and women.

Interestingly, vitamin D, selenium, and fruits and vegetables (and iron for women) are consumed, on average, at rates below the recommendations, according to a recent Swedish dietary study (11). In the international literature, vitamin D and selenium are nutrients with a lower average intake in groups of low SES (17). Thus, the nutrients that would be most expensive to fulfill for a rational consumer are also in several cases the nutrients of which the average intake is below the recommendation. This suggests that cost plays a role in explaining inadequate intake, which is consistent with previous studies (18, 19, 21, 22).

The shadow price analysis also shows how the cost of fulfilling different recommendations has evolved over time. This can be used as a way to better understand the underlying factors of the evolution of the overall MPNFI index. There are large differences in how the cost of adhering to different recommendations has changed over time, as seen in Fig. 3. The cost of meeting the recommended 500 g of fruits and vegetables is becoming relatively cheaper, whereas vitamin D and iron especially are becoming more expensive to obtain. This information can be used for evaluating policy suggestions. From the shadow price analysis, subsidizing fruits and vegetables seems an inefficient way to increase public health. Even though the cost of vegetables does limit the optimal diet in the MP model (i.e. the shadow price is larger than zero), this cost seems to decrease relative to other factors over time (the fruits and vegetables shadow price index shows a very modest increase). Thus, if price is a deterring factor for the consumption of fruits and vegetables, it is expected to become less and less important over time if the trend continues. If, for example, price manipulation (44) should be attempted, fish and shellfish seem a more reasonable selection. These contain high levels of vitamin D and selenium, nutrients that are more expensive to obtain even for a rational and enlightened consumer. Furthermore, policies to increase intake of vitamin D, which is currently below recommended levels, should take into consideration that this nutrient has become increasingly expensive to obtain during the last decades.

Although shadow pricing is clearly a promising technique, it should be remembered that the calculated shadow prices depend on the modeling assumptions, for example, rationality, availability of foods, and what is defined as a palatable diet. Future studies should focus on investigating these effects and to what extent shadow prices are related to actual insufficient intake for individual consumers.

### Methodological limitations

The price levels were estimated from present prices and deflated using the CPI for appropriate food groups. Not relying on the prices of individual foods has its advantages in not putting too much emphasis on individual foods. However, changes within a price group – for example relative price changes between different vegetables – are not reflected in this measure. The validation of volume-adjusted per capita food expenditure offers an indication that the methodology is able to describe how changes in prices of food groups influence diet cost; however, comparison with long-term absolute prices would offer compelling further validation and should be attempted before implementing suggested policies.

The MP diet deviates substantially from the average consumption, as seen from Table 3. The MD diet is much closer to the national average; however, it does suggest a more than doubled intake of fish and eggs. Because the national average is, according to (11), deficient in many nutrients, it is reasonable to expect changes in any attempt to make the diet adhere to recommendations. It should also be emphasized that, although nutritionally fulfilling, the diets are not necessarily ‘healthy’ in all meanings of the word. The MP diet in Table 3 does adhere to the included recommendations, but does so by suggesting a diet with low variety and large consumption of some foods generally considered unhealthy, for example an average consumption of 40 g of ice cream and 14 g of sugar per day. Including more constraints, for example an upper constraint on added sugar, would be an interesting addition. At present the national database (40) does not support this data for the selected foods; however, no major effects on MD or MP cost are expected. Food with high levels of added sugars are not expected to be very nutrient dense or to deliver micronutrients cost-effectively, they could therefore be substituted for by products without added sugar, without influencing cost.

Both modeling approaches suffer from limitations. An advantage of using two models is that they rely to different extents on the quality of different parts of the underlying data. The highly price-sensitive MP model relies more heavily on correct price estimations over time, whereas the MD model relies more on the estimated average intake. That both indexes show similar trends suggests that the methodology is insensitive to small variations in these assumptions.

There are different choices for the nutritional constraints than the ones suggested in Table 2; using other reference values would influence the cost to some degree. A stability analysis shows that the results are relatively insensitive to small changes in the nutritional constraints. Varying the maximum sodium intake level up or down 20% or setting an energy requirement equal to either the lower or the upper constraint level does not influence the final index value more than ±1%.

## Conclusions

The aim of this study was to construct a price index describing how the cost of nutritious diets has evolved over time. The study shows that a rational consumer looking to either minimize price or deviate as little as possible from the average consumption while fulfilling all nutrient recommendations has not experienced a larger cost increase than the general price increase of food in Sweden between 1980 and 2012. Still, the nutrients that are the most costly even for the cost-minimizing rational consumer to obtain (vitamin D, iron, and selenium) are also nutrients with an insufficient average intake in the general population, according to previous studies. Furthermore, some nutrients with insufficient intake (vitamin D and iron) are becoming increasingly expensive to obtain, whereas it has become relatively less expensive to comply with the recommended amount of fruits and vegetables.

The study illustrates how a nutritional index can be used to better understand the relationship between price and nutrients over time and find candidates for nutrients related to nutritional inequality. Such an index is a tool to complement food CPI statistics for monitoring both the price of nutritious diets and the price of fulfilling individual nutritional recommendations over time.
